# Symphonies of Growth: Unveiling the Impact of Sound Waves on Plant Physiology and Productivity

**DOI:** 10.3390/biology13050326

**Published:** 2024-05-07

**Authors:** Mario Pagano, Sonia Del Prete

**Affiliations:** 1 Institute of Research on Terrestrial Ecosystems (IRET), National Research Council (CNR), Via Madonna del Piano 10, Sesto Fiorentino, 50019 Florence, Italy; 2 Institute of Biosciences and Bioresources (IBBR), National Research Council (CNR), Via Pietro Castellino 111, 80131 Naples, Italy; sonia.delprete@ibbr.cnr.it

**Keywords:** sound waves, Plant Acoustic Frequency Technology (PAFT), sound pressure level, Hz, decibel, plants

## Abstract

**Simple Summary:**

The use of sound wave technology on different plant species has revealed that variations in the Hz, sound pressure intensity, treatment time, and type of setup of the sound source significantly impact the plant performance. For example, a study conducted on cotton plants treated by Plant Acoustic Frequency Technology (PAFT) highlighted improvements across various growth parameters. In particular, the treated samples showed increases in the height, leaf area, and number of boll-bearing branches, as well as other plant anatomical parts. In other cases, such as in transgenic rice plants, GUS expression was upregulated or downregulated concerning the Hz employed. This paper presents a complete, rationalized and updated review of the literature on the effects of sound waves on the physiology and growth parameters of sound-treated plants.

**Abstract:**

The application of sound wave technology to different plant species has revealed that variations in the Hz, sound pressure intensity, treatment duration, and type of setup of the sound source significantly impact the plant performance. A study conducted on cotton plants treated with Plant Acoustic Frequency Technology (PAFT) highlighted improvements across various growth metrics. In particular, the treated samples showed increases in the height, size of the fourth expanded leaf from the final one, count of branches carrying bolls, quantity of bolls, and weight of individual bolls. Another study showed how the impact of a 4 kHz sound stimulus positively promoted plant drought tolerance. In other cases, such as in transgenic rice plants, GUS expression was upregulated at 250 Hz but downregulated at 50 Hz. In the same way, sound frequencies have been found to enhance the osmotic potential, with the highest observed in samples treated with frequencies of 0.5 and 0.8 kHz compared to the control. Furthermore, a sound treatment with a frequency of 0.4 kHz and a sound pressure level (SPL) of 106 dB significantly increased the paddy rice germination index, as evidenced by an increase in the stem height and relative fresh weight. This paper presents a complete, rationalized and updated review of the literature on the effects of sound waves on the physiology and growth parameters of sound-treated plants.

## 1. Introduction

For a long time, plants were defined as static and immutable organisms, but this vision is finally changing, with plants described as living beings capable of recognizing their surroundings and responding to a multitude of stimuli by modifying their growth and development. In particular, acoustic perception and communication in some ways represent the last frontier of research. To date, few research studies have been carried out on the effect that sound, differentiated by intensity and frequency, can have on plant growth. Sound is a pressure wave, which is created by a vibrating object transmitted through gases, liquids, and solids. The frequency (Hz), intensity (dB) and timbre are the characteristics by which sound waves are identified. In particular, one complete oscillation of a sound wave is called a cycle. The Hertz (Hz) unit measures the frequency of that cycle. In other words, one Hertz is equal to one cycle per second (1 Hz = 1 cycle s^−1^). The massive variation under typical acoustic pressures necessitates the use of a relative measurement scale rather than an absolute one. These scales employ logarithms to condense the wide dynamic range. In the field of acoustics, the scale about sound pressure is defined so that every factor of ten increase in the amount of energy carried by the wave is represented as a change of 1 bel. However, the bel unit is frequently too huge to be useful. Consequently, the decibel scale (1 × 10^−^^1^ of a bel) is commonly utilized. Subsequently, the Sound Intensity Level (*SIL*) can be expressed as the logarithm of two intensities:

$$SIL\;\left(\mathrm{dB}\right)=10\log\left(\frac{I}{I_{ref}}\right)$$

where *I* represent the intensity of the sound wave and *I_ref_* is a reference intensity. For the intensity of a sound wave in air, the reference intensity is defined to be:

*I_ref_* = 10^−12^ W m^−2^

In air, the intensity decays proportionally to 1/r^2^, where r denotes the distance from the sound source [1]. Furthermore, the speed with which sound propagates depends on the medium it is passing through and is generally directly proportional to the elasticity and inversely proportional to the density [2]. In the acoustic spectrum, the lowest frequency classification is known as infrasound, with a frequency range below approximately 20 Hz. Infrasound has found applications in both diagnostic and therapeutic contexts. Conversely, ultrasound refers to sound waves with frequencies greater than 20,000 Hz and has been extensively utilized in medical practice for more than five decades as a tool for both diagnosis and treatment [3,4,5]. The audible sound that is perceptible by humans has frequencies from 20 to 20,000 Hz, and the role of sound in the animal kingdom is well studied. How plants (as sessile organisms) recognize sound has not been extensively elucidated due to the lack of an organ in plants designed to recognize air vibrations, like eardrums in humans [6]. Recent studies have endeavored to delineate the responses of plants to sound waves [7,8]. However, it is known that plants communicate essentially in two ways: either by sending volatile chemical signals [9] or through the network of fungi that intersects their roots [10]. Plants are in continuous contact with organisms in the environment around them through the production of volatile organic compounds (VOCs). VOCs are real sound producers for plants. In fact, through them, plants communicate with their surroundings, regulating their growth, development, defense, propagation and life cycle to reach the maximum physical form. The VOCs that mediate the communication of plants and other organisms belong to a range of different compound classes, such as fatty acid-derived molecules (including GLVs), terpenoids, benzenoids, and phenylpropanoids, and minor classes such as nitriles, (ald)oximes and sulfides. Fatty acid-derived VOCs (including GLVs) are produced from the C18 unsaturated fatty acids, linoleic and linolenic acid [9]. The highly studied volatile compounds mediate the interaction of plants with other plants and microorganisms [11]. The type of sound communication used by plants is very little known; it would appear that they can produce sound waves at relatively low frequencies such as 50–120 Hz [10]. Moreover, leaves of *Phylodendron* have been observed to spontaneously generate sound waves with frequencies ranging from 50 to 120 Hz. Additionally, plants might have a system comparable to that found in other living organisms, allowing them to absorb and resonate to specific external sound frequencies [12,13]. Limited information exist on the impacts of audible sound on plants. Recent applications of audible sound wave technology to plants, encompassing different phases, such as seed sprouting, callus development, hormone activity, and mechanisms of photosynthesis, and genetic coding, suggest potential benefits. Sound stimulation has been shown to enhance disease resistance and reduce the need for chemical fertilizers and biocides. The impact of sound waves on plant biology is evident: affecting the cell cycle, plant leaf vibrations, and protoplasmic movement in cells [14,15]. While acoustic biology has gained popularity, there remains ambiguity and discrepancies in this field of research, with investigators experimenting with diverse music types and sound frequencies, yielding varying results [16]. The mechanisms responsible for the effects of sound on plants remain, in many cases, undisclosed. Some studies on *Chrysanthemum* seedlings highlighted that sound stimulation may activate stress-induced genes, leading to increased transcription levels [14]. In another way, sound frequency technology has been shown to stimulate leaf stomata opening, enhancing the plant’s uptake of spray fertilizer. Furthermore, sound waves have proven efficiency in facilitating herbicide absorption, allowing for a 50% reduction in herbicide and biocide use on mature weeds. Consequently, sound waves have the potential to decrease the reliance on chemical fertilizers and pesticides [17]. Moreover, the interaction between sound and light is noted, where both of them can transform and stock as energy in chemical form, enhancing the photosynthesis system [18]. This comprehensive review synthesizes the diverse impacts of sound waves on plants, emphasizing gene expression modulation, sonic techniques, metabolic processes, seed germination enhancement, and the PAFT approach. In particular, it aims to provide an updated perspective on how sound waves influence various aspects of plant physiology and growth, encompassing stress-induced gene expression alterations, adjustments in stomatal conductance, and morphological changes. At this point, it is clear that the variety of sounds, of different frequencies and intensities, to which plants are subjected produce different response mechanisms at a genetic, metabolic, morphological level. The objective of this review is to delve deeper into the research on the perception of sound in plants, as this could also have important repercussions in the fields of agriculture and biotechnology. In fact, for example, if you are able to precisely evaluate the state of crop water through acoustic emissions, you could also have more efficient irrigation. Furthermore, sound could also be used to increase the shelf life of products, increase yields or activate plant defenses against pathogens. The view of plants as static organisms is evolving, leading us to consider these organisms as being so ingenious that they can offer significant advantages to the outside world. The method used to select the bibliography sources was based on a search of the relevant literature about the topic employed in this review. In particular, databases such as Scopus, Web of Science, Google Scholar, Semantic Scholar and other online databases were utilized to identify a wide range of scholarly articles relevant to this research topic.

## 2. Exposure to Sound Vibration (SV) Lead to Changes in Gene Expression

**Figure 1:**
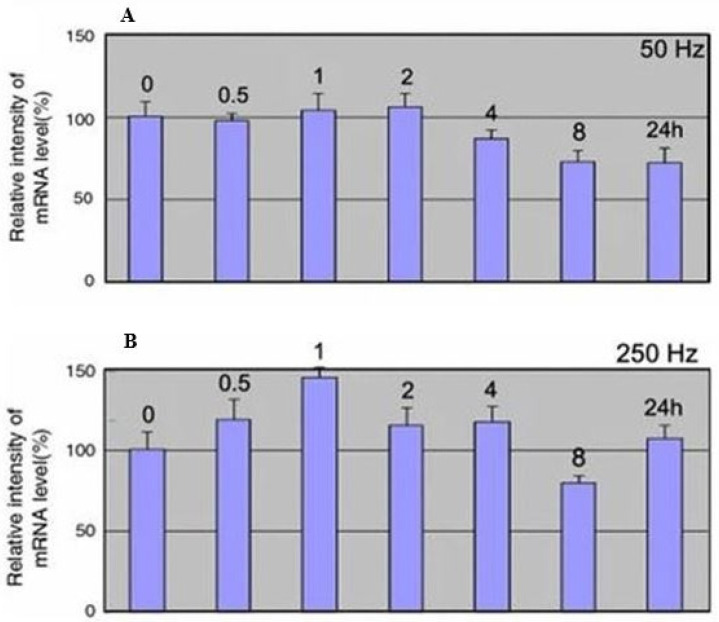
Temporal investigation of ald mRNA expression in rice plants. At the indicated times, RNA was extracted from the untreated control and plants treated at a frequency of 50 and 250 Hz (panel (**A**) and panel (**B**), respectively). GUS expression was upregulated at 250 Hz for 4 h (positive effect) but downregulated at 50 Hz for the same time (negative effect) [23].

Plants are able to pick up sounds and adapt to the environment around them, modifying their growth and development. For example, wind and touch, known as the major mechanical disturbances involving plants, cause physiological and developmental changes in plants, making them more resistant to other mechanical stresses. One of the mechanical stress on plants is sonication. As shown in the literature, sonication could be an advantage for plants. In fact, sonicating tissue during infection with *Agrobacterium*-mediated genetic transformation has been reported to increase the production of small and uniform fissures and channels throughout the plant tissue, which increased the access of *Agrobacterium* [19]. Sonication has been shown to raise the transformation efficiency, particularly in species recalcitrant to transformation. For example, sonication of 60 kHz in 40 s of *Leptadenia pyrotechnica* explants, before the *Agrobacterium* infection, produced about four times more stable transformations than the control [20]. Regarding the consequences of sound, it is reported in the literature that sound stimulation in the *Arabidopsis thaliana* species changes the expression levels of a set of genes known as touch genes (TCH) [21]. The presence of these genes indicates that specific receptors and signal transduction pathways are activated in response to environmental modifications. In the literature, there are various conflicting data on the effects of sound on plants [22]. For example, in the growing *Scendemus obtusiculus* species, it is reported that an audible and low ultrasonic frequency creates difficulties in reproducing the experiments because the precise experimental conditions are not indicated. Furthermore, Mi-Jeong Jeong et al., making use of complex musical sounds and single frequencies, have isolated several sound-induced genes and mRNA analyses have highlighted that a specific modification to the frequency of expression of the gene codes for the cytoplasmic protein aldolase (ald). Specifically, the researchers studied how the ald promoter responds to sound, producing transgenic rice plants harboring a chimeric gene comprising a fusion of the ald promoter and the GUS reporter. In all three transgenic lines, GUS expression was upregulated at 250 Hz for 4 h but downregulated at 50 Hz for the same time (Figure 1) [23].

In 2016, Gosh et al. [21] identified 17 SV-regulated genes (SRGs) that were upregulated by sound vibration treatments in *Arabidopsis*. For different time periods, they analyzed the expression patterns of similar genes after an exposure of 500 Hertz at 80 decibels. Simultaneously, they confirmed the SV-mediated expression of these genes under lighted condition, as many of them were reported to be dark-induced [21]. Moreover, sound has been found to alter epigenetic profiles such as the histone tail modifications of an organism. Several histone modifications, such as H3K36ac, H3K4me3, and H3K36me, are active histone marks promoting gene expression, whereas H3K27me3 is an inactive mark, which may act as a biomarker for specific sound treatment (H3K36ac), and an evolutionary conserved plant histone modification that marks active genes [24,25].

## 3. Sonic Strategies: Plant Drought Resilience and Revealing Signaling Responses

Jeong et al. [26] observed that prior sound vibration (SV) treatment led to an improvement in the relative water content, stomatal conductance, and quantum yield of PSII (Fv/Fm ratio) in *Oryza sativa* affected by drought. Various factors may contribute to the observed response; yet, based on the available evidence, it appears that SV treatment induces a priming or hardening effect. This effect adjusts the physiological conditions within cells, enabling plants to exhibit drought tolerance more rapidly and effectively than untreated plants. The onset of drought primarily results in the accumulation of Reactive Oxygen Species (ROS). The heightened ROS levels trigger a signaling cascade associated with sugar-sensing and Ca^2+^ fluxes, initiating acclimatory responses [27]. In support of this, SV treatments have been observed to impact cellular levels of ROS, sugars, and Ca^2+^. Recent studies have explored the possibility that plants are able to utilize acoustic signals, induced by drought, to communicate with their neighboring plants and prepare them for the problem of water scarcity [28,29]. It is known as the process of drought-induced cavitation. This process leads to the formation of air bubbles in plant xylem, resulting in acoustic emissions upon bursting, continuously recorded on the stems of trees of *Pinus sylvestris* and *Quercus pubescens* [29,30]. Other studies highlighted that the impact of a 4 kHz sound stimulus extends beyond communication, positively influencing drought tolerance. This intervention has been associated with a 40.89% increase in the yield and a 10.3% rise in the soybean protein content [31,32]. Notably, sound frequencies also contributed to an increase in the relative water content of plants experiencing drought stress, with the highest observed in plants treated with 1.5 kHz compared to the control group. Both fresh and turgid weights have been identified as significant factors influencing the leaf water content under drought conditions, as supported by previous studies [33,34,35,36,37,38]. In this way, it is well known that a water deficit can negatively impact the relative water content of plants in drought environments [26]. However, the plant’s ability to survive in drought conditions is contingent on its capacity to either restrict water loss or maintain a high relative water content of more than 60% [37]. Sound frequencies have been found to enhance the osmotic potential, with the highest observed in *Oryza sativa* samples treated with frequencies of 0.5 and 0.8 kHz compared to the control [26]. Moreover, these frequencies have demonstrated a significant increase in the dark-adapted quantum yield (Fv/Fm ratio) during drought stress conditions. The treated plants exhibited an improved dark-adapted quantum yield (Fv/Fm ratio) when compared to the control, particularly at frequencies of ≤0.8 ± 0.045. In a specific experiment involving *Arabidopsis* plants cultivated at 22 °C under an 8/16 h light–dark photoperiod, a one-week treatment with 10 h of 100 dB white noise during the middle of the dark period resulted in heightened drought tolerance. In particular, this treatment showed significantly higher survival rates (24.8% ± 3.81) compared to the untreated plants, where only 13.3% (±3.16) exhibited increased survival [39]. These findings collectively highlight the multifaceted impact of sound treatments on plants, especially when they are experiencing drought stress. High-frequency sound waves, alongside nutrition, have been observed to stimulate the opening of stomata in *Brassica juncea* L. plants [40]. However, at 3 h of exposure, there is a noticeable decline in plant growth, particularly evident with the treatment at a maximum frequency of 5000 Hz [40]. Sound vibrations have the potential to widen the aperture of the stomata, facilitating increased absorption of water and CO_2_, thereby optimizing the photosynthetic process. Consequently, this optimization can promote plant growth and productivity [40].

## 4. Impact on Energetic Metabolism

**Figure 2:**
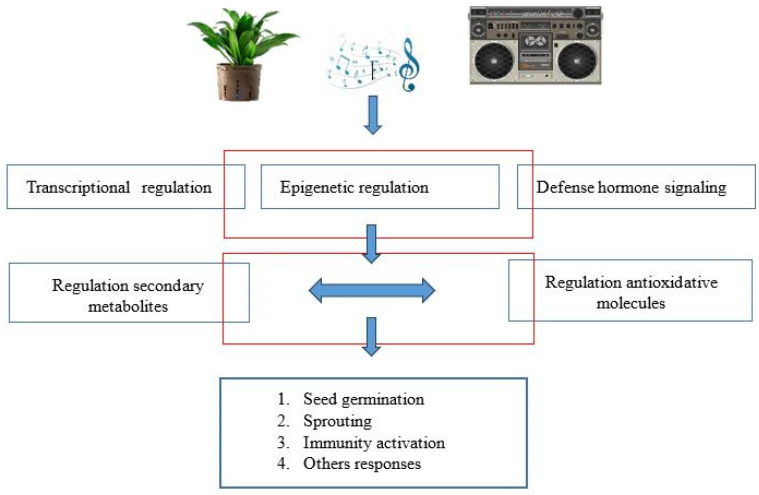
Sound stimulation involves the biosynthetic pathway of secondary metabolites (elaborated from [41]).

The production of secondary metabolites can occur for various reasons, which include the type of sound, the variety of plant species, differentiation in growth, the frequency of the sound, the time of exposure to the sound, etc. Some recent studies have revealed that plants also respond to sound waves. Transcriptional, epigenetic and hormonal signaling changes, which lead to the formation and modulation of secondary metabolites or antioxidant substances, are due to the stimulation of plants by sound waves. In Figure 2, a schematic representation of this process is shown.

For example, in 2003, Qin et al. [42] demonstrated that sound exposure of Chinese cabbage and cucumber in two different growth stages (seedlings, mature plants) in Chinese territory caused a higher production of polyamines compared to the control and a greater absorption of O_2_. This was reflected in the plant with a better growth rate [42]. In 2004, similar results, again in China, were found by Ye et al. in terms of the plant *Taxus chienensis*. The study revealed the effects of a pulsed electric field (PEF) on the studied plant, which led to increased production of secondary metabolites [43]. The production of the intracellular bioactive secondary metabolite Taxuyunnanine C, reactive oxygen species (ROS) and phenols, all classified as defense responses in plant cells, increased after exposure to 30 min of a PEF. It is reported in the literature that sound regulates Ca^2+^ and ROS signaling pathways to modulate plant cells [44]. Indeed, Jung et al. reported that Ca^2+^ ions flowed into the cytosol from the plant’s outer membrane through exposure to a 1 kHz sound wave. This indicated that Ca^2+^ and ROS species can also serve as messengers against environmental stresses, such as microbial pathogens [6]. Furthermore, at very high intensity levels, sound waves induce the biosynthesis of antioxidant substances, which increases the absorption of oxygen in plant cells and also the growth of plants. These physiological changes may include the shorter germination period of seeds, faster differentiation of healing tissues, and better growth of seedlings or mature plants. In fact, a particular study in *Arabidopsis thaliana* showed that sound waves (100 and 100 + 9 kHz) can improve plant growth by regulating genes involved in the metabolism of functional substances at a root level [45]. Flavonoids and isoflavonoids are considered secondary metabolites involved in antioxidant, immunomodulatory, antiviral, anticancer and antiaging properties. The quality of vegetables and plants is improved by the content of these flavonoids. In a study, exposure to different sound frequencies was found to increase the flavonoid content in *Medicago sativa*, *Brassica oleracea* and *Raphanus sativus* [46]. These results were in accordance with the qPCR profiles of the flavonoid biosynthesis genes. DPPH (1,1-diphenyl-2-picrylhydrazyl) and FRAP (ferric-reducing antioxidant power) assays supported the increased antioxidative characteristics in the sound-treated sprouts. Cell suspension cultures of *Genista tinctoria* significantly enhanced the genistin (isoflavone) content after ultrasound (35 kHz) exposure [47]. Recently, the researchers Azgomi et al. conducted a study in Iran examining the impact of music and noise on plant flavor. They found that all the acoustic treatments promoted germination, growth, and biomass accumulation. Furthermore, these treatments enhanced the activity of the phenylalanine ammonia-lyase (PAL) enzyme and increased the total phenolic concentrations. Notably, the highest concentration of flavonoids was strongly correlated with an increase in the expression of the basic leucine zipper transcription factor gene in *Satureja hortensis* L. [48].

## 5. Seed Germination

Several studies have investigated the impact of mechanical vibration, including the frequency and amplitude, on seed germination. For example, in *Cucumis sativa* and *Oryza sativa*, seed germination was promoted with a frequency of 50 Hz. In the same way, in *Arabidopsis thaliana*, providing a fixed amplitude of vibration at 0.42 mm and frequencies above 70 Hz resulted in an increased rate of seed germination [49,50,51,52]. In other experiences, the application of sound wave treatment notably enhanced the germination process of both rice and cucumber (*Cucumis sativus*) seeds [45,51,53]. Wang et al. [14] emphasized the impact of sound waves on paddy rice seeds, revealing that at a sound frequency of 0.4 kHz and SPL of 106 dB, significant increases were observed in the germination rate, stem length, growth rate of fresh weight (*p* < 0.01), root system performance, and cell membrane permeability (*p* < 0.05). However, exceeding 4 kHz or 111 dB in sound wave stimulation resulted in the inhibition of paddy rice seed growth [10].

## 6. Plant Acoustic Frequency Technology (PAFT) and Agri-Wave Technology

### 6.1. The PAFT Approach

**Figure 3:**
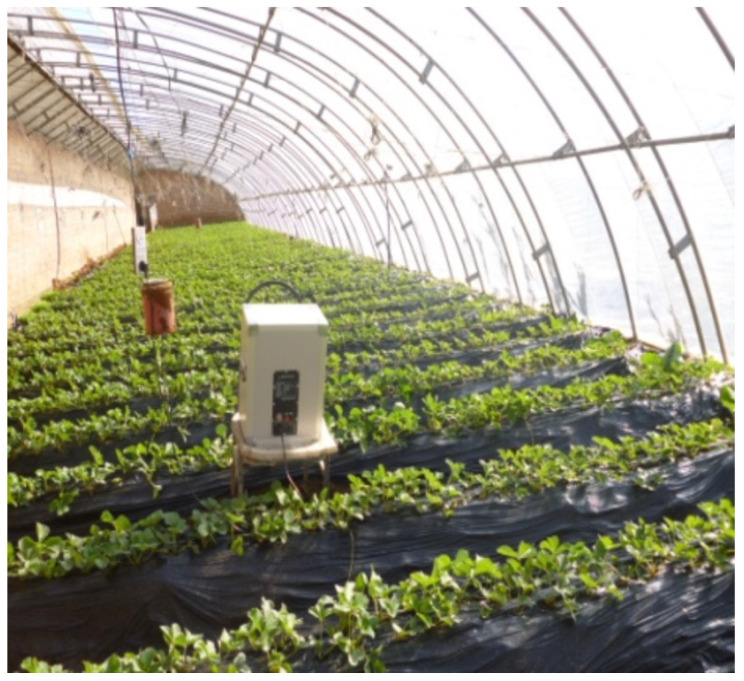
Representation of the Chinese solar greenhouses in Beijing, China (latitude 40.18° N, longitude 116.47° E), featuring cultivation of *Fragaria ananassa*. The image also depicts the Plant Acoustic Frequency Technology (PAFT) generator developed by the Qingdao Physical Agricultural Engineering Research Center in China [61].

The Plant Acoustic Frequency Technology (PAFT) generator (Figure 3) manufactured by the Qingdao Physical Agricultural Engineering Research Center in China has eight variable frequency levels from 0.06 to 2 kHz and sound pressure levels (SPLs) from 50 to 120 dB for a distance about 50–100 m. PAFT produces an intermittent pulse of sound wave frequencies [10]. Research indicates that acoustic frequency treatment has the potential to enhance the function of the photosystem II reaction site, improve electron transport, and increase the photochemical efficiency of PS II [10,54,55,56]. Additionally, this treatment has been found to stimulate the production of endogenous hormones, including IAA, GA, and ZR, leading to increased hormone levels in various vegetables, such as cucumber, tomato, muskmelon, cowpea, and eggplant [10,18,57,58]. Notably, strawberries treated with PAFT exhibited stronger growth compared to the control group, with deeper green leaves and earlier blossoming and fruit-bearing by approximately one week. Furthermore, the photosynthetic rate of treated strawberries significantly improved (*p* < 0.05). Although the treatment enhanced strawberry resilience against disease and pests, its impact on yield was minimal [10,59]. A study conducted on cotton plants treated with PAFT revealed that various growth parameters experienced improvements. In particular, the treated samples exhibited a 1.7% increase in height, a 5.2% increase in the width of the fourth fully expanded leaf from the terminal one, a 1.1% increase in the number of branches bearing bolls, a 9.2% increase in the number of bolls, and a 3.3% increase in the weight of individual bolls [10]. Additionally, the average yield of treated cotton showed a significant increase of 12.7%. Furthermore, it was noted that the effectiveness of PAFT, when employing four speakers, on cotton plants was contingent upon the distance from the sound source in different directions. The minimum yield increase of 5.2% was observed at a distance of approximately 30 m from the PAFT, with a sound pressure level (SPL) ranging from 75 to 110 decibels, while the highest yield increase of 18.6% occurred at distances ranging between 30 and 60 m with an SPL of 70–75 decibels. However, beyond a distance of 150 m, no significant effect on yield was observed [10,60]. Other research highlighted that the average increase in the rice yield was 25.0% in pot experiments and 5.7% in open-field conditions, while the wheat yield increased by an average of 17.0% when subjected to the PAFT generator [10]. Rahman et al. [32] investigated, by a PAFT approach, the sound effects of *Dundubia manifera* on the stomatal density and stomatal index. The sound frequency was adjusted to 4000 Hz using Adobe Audition CS6 and applied for 30 min daily over 28 days. The results indicate that exposure to sound waves affects the stomatal density and index. Specifically, the stomatal density decreased by 89% in the treated plants (from 97,506 to 51,428 stomata/mm^2^) compared to the control plants. Conversely, the stomatal index increased by 41% in the treated plants (from 0.194 m to 0.274 m) compared to the control plants. These findings suggest that stomatal manipulation through sound waves can have significant positive impacts on plants, including enhanced photosynthesis rates, improved immune responses, resilience to climate change, and increased crop yields [32]. Hassanien et al. [61] showed that sound treatment notably accelerated the blooming and flowering rates in *Fragaria ananassa* (strawberry) plants. The highest concentration of ABA was observed in the leaves of the treated plants after 90 days of exposure for 5 h daily. Conversely, the mean IAA levels in the leaves of the control plants were slightly higher than those of the treated plants after 90 days of exposure for 5 and 2 h.

### 6.2. Agri-Wave Technology

Agri-wave technology involves radiating discontinuous impulses of acoustic waves employing PAFT and applying a microelement fertilizer mixture to the leaves every other week [10,62]. Researchers conducted a study to explore how agri-wave technology influences the plant meridian system, aiming to enhance the plant yield and quality. The outcomes indicated a significant enhancement of tomato growth, with the fresh mass of the branches, stems, and foliage being notably higher (59.5%, *p* < 0.001) compared to the untreated population. Moreover, the technology accelerated tomato ripening, boosted production (13.9%, *p* < 0.001), and increased the overall quality. Similarly, the application of agri-wave technology to spinach led to heightened growth rates and an amplified yield of the spinach crop. Specifically, the spinach subjected to treatment showed a substantial increase in yield of 22.7% and 22.2%, alongside a remarkable boost in the sugar content of 37.5% and significant elevations in vitamins A, C, and B, escalating by 35.6%, 41.7%, and 40.0%, respectively. In greenhouse experiments, the average weight of three varieties of lettuce treated with agri-wave technology surpassed that of the control group by 44.1% [10,16].

## 7. Conclusions

Studies have demonstrated that various plant species exhibit several reactions to stimulation by acoustic waves in distinct ways. In this way, acoustic frequency technology can promote plant performance in terms of the growth, yield quality and other parameters. Furthermore, an investigation involving cotton plants subjected to PAFT demonstrated enhancements across multiple growth parameters. Notably, the treated samples exhibited increases in height, the size of the fourth fully developed leaf from the final one, the quantity of branches bearing bolls, the count of bolls, and the mass of individual bolls of 1.7%, 5.2%, 1.1%, 9.2%, and 3.3%, respectively. In other research, experiences highlighted how a 4 kHz sound stimulus can positively affect plant drought tolerance, transcending communication. In transgenic rice, for example, GUS expression increases at 250 Hz but decreases at 50 Hz. Sound frequencies boost the osmotic potential, notably at 0.5 and 0.8 kHz, compared to controls. Treatment at 0.4 kHz with 106 dB SPL significantly enhances the paddy rice germination index, as evidenced by the heightened stem height and fresh weight. In addition, it might be useful to know that sound could also be used to increase defenses against pathogens. However, this mechanism is not yet well studied. In fact, we know that although plants do not possess an adaptive immune system like vertebrates, they can launch specific, self-tolerant immune responses and establish immune memory. We know that to promote virulence, pathogens inject effector molecules that target conserved immune signaling hubs in the plant cell. In response, plants have evolved resistance (R) proteins that sense effector-induced perturbations in these hubs, providing the ability to specifically recognize large numbers of pathogens with similar infection strategies through fewer R proteins. These R proteins can specifically recognize pathogen effectors and trigger a more potent and long-lasting immune response known as ETI. This paper aims to present a comprehensive and updated review of the literature concerning the influence of sound waves on plant physiology and growth factors. In particular, the paper highlights the mechanism through which sound influences plants at the cellular level and affects the physiological performance. Overall, this comprehensive review underscores the need for continued research into the effects of sound waves on plant physiology and growth parameters. By elucidating the cellular mechanisms through which sound influences plants, future studies can unlock new strategies for optimizing agricultural practices, improving crop resilience, and ultimately contributing to global food security. Finally, this review provides a comprehensive summary of the frequencies of sound waves that can be utilized for novel agricultural practices and biotechnological treatments of plants.

## Data Availability

Not applicable.
